# The Relationships between Polymorphisms in Genes Encoding the Growth Factors TGF-β1, PDGFB, EGF, bFGF and VEGF-A and the Restenosis Process in Patients with Stable Coronary Artery Disease Treated with Bare Metal Stent

**DOI:** 10.1371/journal.pone.0150500

**Published:** 2016-03-01

**Authors:** Tadeusz Osadnik, Joanna Katarzyna Strzelczyk, Rafał Reguła, Kamil Bujak, Martyna Fronczek, Małgorzata Gonera, Marcin Gawlita, Jarosław Wasilewski, Andrzej Lekston, Anna Kurek, Marek Gierlotka, Przemysław Trzeciak, Michał Hawranek, Zofia Ostrowska, Andrzej Wiczkowski, Lech Poloński, Mariusz Gąsior

**Affiliations:** 1 Third Department of Cardiology, School of Medicine with the Division of Dentistry, Medical University of Silesia, Zabrze, Poland; 2 Genomics Laboratory, Kardio-Med Silesia Science and Technology Park, Zabrze, Poland; 3 Department of Medical and Molecular Biology, School of Medicine with the Division of Dentistry, Medical University of Silesia, Zabrze, Poland; University of Birmingham, UNITED KINGDOM

## Abstract

**Background:**

Neointima forming after stent implantation consists of vascular smooth muscle cells (VSMCs) in 90%. Growth factors TGF-β1, PDGFB, EGF, bFGF and VEGF-A play an important role in VSMC proliferation and migration to the tunica intima after arterial wall injury. The aim of this paper was an analysis of functional polymorphisms in genes encoding TGF-β1, PDGFB, EGF, bFGF and VEGF-A in relation to in-stent restenosis (ISR).

**Materials and Methods:**

265 patients with a stable coronary artery disease (SCAD) hospitalized in our center in the years 2007–2011 were included in the study. All patients underwent stent implantation at admission to the hospital and had another coronary angiography performed due to recurrence of the ailments or a positive result of the test assessing the coronary flow reserve. Angiographically significant ISR was defined as stenosis >50% in the stented coronary artery segment. The patients were divided into two groups–with angiographically significant ISR (n = 53) and without significant ISR (n = 212). Additionally, the assessment of late lumen loss (LLL) in vessel was performed. *EGF* rs4444903 polymorphism was genotyped using the PCR-RFLP method whilst rs1800470 (*TGFB1*), rs2285094 (*PDGFB*) rs308395 (*bFGF*) and rs699947 (*VEGF-A*) were determined using the TaqMan method.

**Results:**

Angiographically significant ISR was significantly less frequently observed in the group of patients with the A/A genotype of rs1800470 polymorphism (*TGFB1*) versus patients with A/G and G/G genotypes. In the multivariable analysis, LLL was significantly lower in patients with the A/A genotype of rs1800470 (*TGFB1*) versus those with the A/G and G/G genotypes and higher in patients with the A/A genotype of the *VEGF-A* polymorphism versus the A/C and C/C genotypes. The C/C genotype of rs2285094 (*PDGFB*) was associated with greater LLL compared to C/T heterozygotes and T/T homozygotes.

**Conclusions:**

The polymorphisms rs1800470, rs2285094 and rs6999447 of the *TGFB1*, *PDGFB* and *VEGF-A* genes, respectively, are associated with LLL in patients with SCAD treated by PCI with a metal stent implantation.

## Background

After percutaneous coronary intervention (PCI), neutrophils and leukocytes accumulate in the arterial wall, and an increase in the levels of inflammatory response mediators is observed. Histological analyses have revealed that in patients with a metal stent implanted, during the first week after the procedure, the neointima consists of 60% smooth muscle cells (vascular smooth muscle cells [VSMCs]) and 30% neutrophils [1]. In successive weeks following the procedure, the neutrophil ratio decreases, and VSMCs represent over 90% of neointima cells [1]. Through a series of processes, mechanical injuries of vessel walls result in VSMC activation and proliferation and a phenotype change from contractile to proliferative and secretory [2]. The increase in VSMC proliferation leads to gradual narrowing of the vessel lumen (in-stent restenosis [ISR]). Growth factors, including transforming growth factor beta 1 (TGF-β1), platelet-derived growth factor beta (PDGFB) [3], epidermal growth factor (EGF) [4] and basic fibroblast growth factor (bFGF), play an important role in smooth muscle cell (SMC) proliferation and migration to the tunica intima [3]. Numerous studies of restenosis have also indicated a role of vascular endothelial growth factor A (VEGF-A) in this phenomenon [5–8].

The effects of TGF-β1 on VSMCs and its role in the restenosis process have been the subject of numerous studies [9–12]. Some reports indicate that TGF-β1 levels, and possibly activity, may depend on genetic factors [13], including the rs1800470 polymorphism. A recently published study examined the relationship between *TGFB1* polymorphisms and restenosis in the Mestizo population [14].

The association of the rs2285094 polymorphism of the *PDGFB* gene, which is located in an intron near an mRNA splice site, with the development of type 1 diabetes [15], IgA nephropathy [16], and scleroderma [17] has been analysed. The rs308395 polymorphism within the *bFGF* gene promoter may influence transcription factors binding, and thus *bFGF* expression [18,19]. The rs4444903 polymorphism (A61G) within the *EGF* gene promoter region is associated with EGF levels and various neoplastic diseases [20,21]. The rs699947 polymorphism of the *VEGF-A* gene is associated with a higher risk of developing certain neoplastic diseases [22] and, in cardiology, with the development of collateral circulation [23] or a response to anti-hypertensive therapy [24]. Although the roles of these growth factors [3–8] in restenosis are known, only one paper has described the role of *TGFB1* gene polymorphisms in restenosis, whereas the roles of functional polymorphisms in the genes encoding PDGFB, bFGF, EGF and VEGF-A in restenosis have not been studied. The aim of this paper was to analyse the relationship between polymorphisms in the *TGFB1*, *PDGFB*, *bFGF*, *EGF* and *VEGF-A* genes and ISR in patients with stable coronary artery disease (SCAD).

## Materials and Methods

The methods were described previously [25]. Briefly, we enrolled 265 patients with 322 lesions subjected to implantation of at least one bare metal stent and who had subsequent coronary angiography performed because of the recurrence of angina symptoms or a positive result of non-invasive cardiac stress tests. Quantitative coronary angiography (QCA) was used to assess minimal lumen diameter, percent of lumen stenosis and vessel diameter before and after stent implantation and during subsequent coronary angiography. Significant restenosis was defined as the narrowing of the vessel lumen by >50% within or up to 5 mm of the previously implanted stent. Late lumen loss (LLL) was calculated by subtracting the diameter, in millimetres, of the stented segment measured in the follow-up coronary angiography from the vessel lumen measured directly after the stenting procedure. The cardiologists performing the QCA analyses were blinded to the results of the researchers performing the genotyping analysis and vice versa.

### Genetic studies

DNA was extracted from blood samples using the GeneMatrix Quick Blood DNA Purification Kit (EUR_X_, Poland) according to the manufacturer’s instructions. High-quality DNA was extracted from all samples, with an average yield of 51.7 μg (range 5–460 μg).

The *EGF* gene was genotyped using the PCR-RFLP (*Polymerase Chain Reaction—Restriction Fragment Length Polymorphism)* method, whereas the *PDGFB*, *VEGF-A*, *TGFB1* and *bFGF* genes were genotyped using 5' exonuclease TaqMan genotyping assays.

#### Genotyping of the *EGF* gene using PCR-RFLP

The *EGF* gene was genotyped using the PCR-RFLP method using the primers described by Shahbazi et al. [26].

PCR reaction mixtures with a final volume of 25 μl contained the following: 2 μl of genomic DNA, 0.2 μl of 25 mM dNTP Mix (containing dATP, dCTP, dGTP, and dTTP), 1.2 μl of 10 μM forward and reverse primers (Genomed, Poland), 2.5 μl of DyNAzyme Buffer (Thermo Scientific, USA), 0.3 μl of DyNAzyme II DNA Polymerase (2 U/μl; Thermo Scientific, USA) and 17.6 μl of molecular grade water (Qiagen, Germany). The following primers were used: 5'-TGTCACTAAAGGAAAGGAGGT-3' in the forward direction and 5'-TTCACAGAGTTTAACAGCCC-3’ in the reverse direction. The PCR reaction was performed in a Mastercycler Personal thermocycler (Eppendorf, Germany), and the conditions were 5 min of initial denaturation at 95°C, followed by 35 cycles of denaturation at 95°C for 45 s, annealing at 57°C for 45 s and extension at 72°C for 45 s and a final extension at 72°C for 10 min. The length of the amplified *EGF* fragment was 242 bp. The PCR product was digested by *Alu*I to type the *EGF* 61A/G polymorphism by RFLP analysis. The PCR product was digested at 37°C for 12 h in a Mastercycler Personal thermocycler (Eppendorf, Germany) in a solution containing 5 μl of PCR product, 1 μl of *Alu*I restriction enzyme (10 U/μL, Thermo Scientific, USA), 1 μl of 10X Buffer Tango (Thermo Scientific, USA), and 3 μl of molecular grade water (Qiagen, Germany) per reaction. Then, *Alu*I was inactivated by incubation at 65°C for 20 min. The digestion products of *EGF* were separated on 2% agarose gel (Sigma, USA), stained with ethidium bromide (Serva, Germany), and photographed on a UV transilluminator. The sizes of the fragments were compared to the positions of the molecular weight marker (GeneRuler^TM^ 100 bp DNA Ladder, MBI Fermentas, Lithuania). *Alu*I cleaved the 242-bp PCR product into fragments of 15, 34 and 193 bp fragments for GG homozygotes and 15, 34, 91 and 102 bp for AA homozygotes.

#### Genotyping the PDGFB, bFGF, EGF and TGFB1 genes using the TaqMan assay

To identify single nucleotide polymorphisms (SNPs) of the *PDGFB*, *VEGF-A*, *bFGF*, and *TGFB1* genes, 5' exonuclease TaqMan genotyping assays on a 7300 Real-Time PCR System and SDS 1.4 Allelic Discrimination software (Applied Biosystems, USA) were used. The TaqMan genotyping assays used the 5’ nuclease analysis to amplify and detect specific SNP alleles. Each assay contained two primers and two TaqMan MGB probes to detect alleles. The MGB probes consisted of a reporter dye: VIC dye at the 5’ end of the allele 1 probe, 6FAM dye linked to the 5’ end of the allele 2 probe, a minor groove binder, and a non-fluorescent quencher (NFQ) at the 3’ end of each probe. During the data analysis, an increase in VIC dye fluorescence was observed for homozygosity of allele 1, whereas an increase in 6FAM dye fluorescence was observed for homozygosity of allele 2. High fluorescence of both dyes was observed when both alleles were heterozygous. The examined polymorphisms of the *TGFB1*, *PDGFB*, *VEGF-A* and *bFGF* genes are presented in Table 1.

**Table 1:** The sequences of VIC and FAM probes used for polymorphism detection.

Gene	Polymorphism	Nucleotide base	Context sequence [vic/fam]
*TGFB1*	rs1800470	A/G	TAGCCACAGCAGCGGTAGCAGCAGC[A/G]GCAGCAGCCGCAGCCCGGAGGGCGG
*PDGFB*	rs2285094	T/C	CAACCAATAGAGGGGCCAATAGAAC[T/C]GCCCAGCTGAGCCCAGTCAACCCCC
*bFGF*	rs308395	C/G	CTCTTCTATGGCCTACTTTCTACTG[C/G]TATTTGTGTTACTCATGCTACCCAT
*VEGF-A*	rs699947	A/C	GCCAGCTGTAGGCCAGACCCTGGCA[A/C]GATCTGGGTGGATAATCAGACTGAC

Abbreviations: *TGFB1 –transforming growth factor beta 1*, *PDGFB—platelet-derived growth factor beta polypeptide*, *bFGF–basic fibroblast growth factor*, *VEGF-A–vascular endothelial growth factor A*.

The PCR reaction contained 5 ng of genomic DNA, 2x TaqMan Genotyping Master Mix (No AmpErase UNG), 900 nM of each primer and 200 nM of each probe (Applied Biosystems, USA). PCR was performed at 95°C for 10 min and for 40 cycles of 95°C for 15 s and 60°C for 60 s. All assays were performed in 96-well arrays, and each plate contained controls. After cycling, end-point fluorescence was measured on a Real-Time PCR instrument, and genotype was verified using the allelic discrimination analysis module. Genotyping of 10% of the samples was performed for quality control, with complete congruence. We established the genotypes of rs2285094 (*TGFB1*) for 259 (97.7%) patients, rs699947 (*VEGF-A*) and rs308395 (*bFGF*) for 264 patients (99.6%), and rs2285094 (*PDGFB*) and rs4444903 (*EGF*) for all patients.

### Statistical analysis

Continuous and categorical variables were compared between the ISR and no-ISR groups using U-Mann-Whitney and Fisher’s exact tests, respectively. Continuous variables are presented as median and interquartile ranges. Categorical variables are presented as percentages. The Chi-square test was used to determine genotype consistency with the Hardy Weinberg Equilibrium (HWE). The associations between restenosis risk and the analysed polymorphisms were analysed using the following genetic models: dominant, recessive and log-additive for *PDGFB*, *EGF*, *TGFB1* and *VEGF-A* and dominant only for *bFGF* due to the low number of minor homozygotes. Similar to the methodology used by us [25] and others [27,28], these models were used for patients (n = 265) and adjusted for clinical variables that reached a p-value of <0.3 when comparing patients with and without significant ISR (Table 2). Additionally, following the correction of angiographic data that reached p<0.3 in the group comparison (Table 3), the effect of the considered polymorphisms on LLL (a continuous variable) was analysed. p<0.05 was accepted as the threshold of statistical significance. Analyses were completed using NCSS (Number Crunching Statistical Systems, Kayasville USA) software [29] and with the SNPassoc package in the R environment [30].

**Table 2:** Clinical characteristics of the study population.

Variable	Without ISR, n = 212	With ISR, n = 53	P value
Age [years]	64 [57–71]	61 [56–69]	0.29
Male sex	145 (68.4)	40 (75.5)	0.32
Hypertension	145 (68.4)	33(62.3)	0.52
Heart Failure	48 (22.6)	14 (26.4)	0.56
Atrial fibrillation	35 (16.5)	8 (15.1)	0.80
Hypercholesterolemia	112 (52.8)	30 (56.6)	0.62
Previous MI	111 (52.4)	37 (69.8)	0.02
Previous CABG	20 (9.4)	8 (15.1)	0.23
Previous cardiac arrest	7 (3.3)	2 (3.8)	0.99
Diabetes	61 (28.8)	12 (22.6)	0.37
Peripheral Vascular Disease	12 (5.7)	4 (7.6)	0.85
Previous Stroke	6 (2.8)	2 (3.8)	0.99
COPD	15 (7.1)	2 (3.8)	0.38
Family History of CHD	17 (8.0)	5 (9.4)	0.49
Current Smoker	20 (9.4)	2 (3.8)	0.66
Previous Smoker	67 (31.6)	23 (43.4)	0.11
Ejection Fraction [%]	48 [40–55]]	49 [34–51]	0.17
Creatinine [μmol/l]	81 [67–94]	76 [67–85]	0.16
Hemoglobin [mmol/l]	8.7 [8.1–9.2]	8.9 [8.1–9.6]	0.07
Aspirin	207 (97.6%)	53 (100)	0.57
Thienopirydynes	212 (100)	53 (100)	-
Beta blocker	201 (94.8)	51 (96.2)	0.94
ACE-i/ARB	198 (93.4)	50 (94.3)	0.99
Statins	200 (94.3)	49 (92.5)	0.85
Time to coronary angiography [days]	659 [170–1286]	444 [140–1055]	0.25

Continuous variables are presented as median [interquartile range]. Dichotomic variables are presented as number of patients (percentage).

Abbreviations: MI–myocardial infarction, CABG–coronary artery bypass graft surgery, COPD–chronic obstructive pulmonary disease, ACE-I–angiotensin converting enzyme inhibitors, ARB–angiotensin receptor blockers.

**Table 3:** Angiographic characteristic of the coronary lesions treated (n = 322).

Variable	Without ISR, n = 267	With ISR, n = 55	P Value
Artery treated			0.08
LM	8 (3.0)	2 (3.64)	
LAD/D1	82 (30.7)	17 (30.9)	
Cx/OM/IM	91 (34.1)	10 (18.2)	
RCA/PDA/PLA	86 (32.2)	26 (47.3)	
Lesion length [mm]	9.5 [7.1–14.1]	12.9 [8.1–19.2]	0.01
Total stent length per lesion [mm]	16 [13–22]	20 [15–29]	0.002
Lesion type			0.03
A/B1	183 (68.5)	29 (52.3)	
B2/C	84 (31.5)	26 (47.7)	
Vessel diameter [mm]	2.8 [2.4–3.2]	2.5 [2.1–2.8]	0.0001
Vessel diameter <2.5mm	87 (32.6)	29 (52.7)	0.01
Stent underexpansion	4 (1.5)	1 (1.82)	0.86
Dissection	9 (3.4)	6 (10.9)	0.02
Ostial lesion	7(2.62)	7(12.7)	0.01
Bifurcation	23 (8.6)	7 (12.7)	0.34
Predilatation	142 (53.2)	42 (73.4)	0.002
Postdilatation	24 (9.0)	4 (7.3)	0.68
Percent lumen stenosis [%]	91 [85–95]	92 [86–97]	0.14
Minimal Lumen Diameter (post stent) [mm]	2.8 [2.5–3.1]	2.5 [2.3–2.8]	0.001
Minimal Lumen Diameter (follow up) [mm]	2.5 [2.1–2.9]	0.5 [0.3–0.9]	<0.0001

Continuous variables are presented as median [interquartile range]. Dichotomic variables are presented as number of patients (percentage).

Abbreviations: LM–Left main, LAD–left anterior descending artery, D1 – 1^st^ diagonal branch, Cx–circumflex artery, OM–obtuse marginal branch, IM–intermediate branch, RCA–right coronary artery, PDA–posterior descending artery, PLA–posterolateral artery.

The study was approved by the Ethics Committee of the Silesian Medical Chamber, Katowice, Poland. All patients gave their signed consent to participation.

## Results

### Clinical and angiographic characteristics

The ISR patients and no-ISR patients were similar in terms of age, sex, and comorbidities, with the exception of a more frequent history of myocardial infarction in the ISR group (Table 2). The treatment recommended on discharge was also similar in both groups. Post-stenting lesions in which angiographically significant ISR developed most frequently were complex lesions, according to the AHA/ACC classification [31]. Lesions in which ISR developed were also longer and more frequently located ostially, and the procedure itself was more frequently complicated by dissection. Despite more frequent ostial location, the vessels in which significant ISR developed were smaller in diameter than lesions in which significant ISR did not develop after stenting (Table 3).

### Genetic analysis results

The observed frequencies of polymorphisms conformed to HWE for the *PDGFB*, *bFGF*, *VEGF-A* and *TGFB1* genes, but a deviation from HWE was observed for the distribution of the polymorphism of the *EGF* gene (p = 0.007). Angiographically significant ISR was significantly less frequent in individuals with the A/A genotype of rs180047 (*TGFB1*) versus individuals with the A/G and G/G genotypes (Table 4). Restenosis frequency did not differ for individual genotypes of other analysed polymorphisms. The frequencies of rarer alleles of the analysed polymorphisms were similar to those observed in European populations (Table 4).

**Table 4:** Distribution of the genotypes of the analysed polymorphisms and the frequency of restenosis in relation to genotypes in the analysed cohort.

Gene/Polymorphism		Homozygous major	Heterozygous	Homozygous minor	P value	MAF	MAF EU population [32]
*TGFB1* (rs1800470)	Genotype frequency, n(%)	83 (32)	122 (47.1)	54 (20.9)		44%	38%
	Restenosis, n (%)	11 (13.3)	31 (25.4)	10 (18.5)	0.09		
*PDGFB* (rs2285094)	Genotype frequency, n (%)	37 (14.0)	124 (46.8)	104 (39.2)		47%	46%
	Restenosis, n (%)	10 (27.0)	21 (16.9)	22 (21.2)	0.38		
*EGF* (rs4444903)	Genotype frequency, n(%)	94 (35.5)	109 (41.1)	62 (23.4)		44%	39%
	Restenosis, n (%)	19 (19.1)	22 (20.2)	13 (21.0)	0.98		
*bFGF* (rs308395)	Genotype frequency, n(%)	210 (79.5)	51 (19.3)	3 (1.2)		11%	16%
	Restenosis, n (%)	42 (20.0)	10 (19.6)	1 (33.3)	0.71		
*VEGF-A* (rs699947)	Genotype frequency, n(%)	77 (29.2)	123 (46.6)	64 (24.2)		48%	50%
	Restenosis, n (%)	19 (24.7)	22 (17.9)	12 (18.8)	0.48		

Abbreviations: EU–European, *TGFB1 –transforming growth factor beta 1*, *PDGFB—platelet-derived growth factor beta polypeptide*, *EGF–epidermal growth factor*, *bFGF–basic fibroblast growth factor*, *VEGF-A–vascular endothelial growth factor A*.

### Angiographically significant ISR–multivariable analysis

The multivariable analysis that adjusted for clinical variables indicated that the A/A genotype of rs1800470 was related to a lower risk of significant ISR in the dominant model. Rs2285094 rs4444903, rs308395, and rs699947 were not associated with a risk of angiographically significant ISR (Table 5).

**Table 5:** Association of genotype with angiographically significant restenosis.

Gene/Polymorphism	Dominant Model; OR (95% CI)	P	Recessive Mode; OR (95% CI)	P	Log additive model; OR (95% CI)	P
***TGFB1* genotype**	**A/A (ref.) vs. (A/G + G/G)**		**(A/A + A/G) (ref.) vs. G/G**		**Per each G allele**	
OR unadjusted	1.99 (0.96–4.10)	0.05	0.88 (0.41–1.90)	0.75	1.25 (0.82–1.91)	0.30
OR adjusted	2.27 (1.06–4.87)	0.03	0.94 (0.42–2.08)	0.87	1.35 (0.86–2.10)	0.19
***PDGFB* genotype**	**T/T (ref.) vs. (T/C + C/C)**		**(T/T + T/C) (ref.) vs. (C/C)**		**Per each C allele**	
OR unadjusted	0.89 (0.48–1.64)	0.71	1.59 (0.72–3.54)	0.26	1.07 (0.69–1.66)	0.75
OR adjusted	0.87 (0.46–1.64)	0.67	1.44 (0.63–3.30)	0.40	1.03 (0.66–1.62)	0.89
***EGF* genotype**	**A/A (ref.) vs. (A/G + G/G)**		**(A/A + A/G) (ref.) vs. G/G**		**Per each G allele**	
OR unadjusted	1.09 (0.58–2.05)	0.80	1.08 (0.54–2.18)	0.83	1.06 (0.71–1.57)	0.78
OR adjusted	1.02 (0.53–1.97)	0.94	0.94 (0.45–1.95)	0.86	0.99 (0.66–1.49)	0.95
***bFGF* genotype**	**C/C (ref.) vs. (C/G + G/G)**					
OR unadjusted	1.02 (0.49–2.15)	0.95	*	*	*	*
OR adjusted	1.29 (0.59–2.81)	0.53	*	*	*	*
***VEGF-A* genotype**	**A/A (ref.) vs. (A/C + C/C)**		**(A/A + A/C) vs. C/C**		**Per each C allele**	
OR unadjusted	0.68 (0.36–1.28)	0.24	0.89 (0.44–1.83)	0.76	0.82 (0.54–1.25)	0.35
OR adjusted	0.69 (0.36–1.34)	0.28	0.96 (0.46–2.00)	0.91	0.85 (0.55–1.30)	0.45

* not calculated because of low number of minor homozygotes.

The models were adjusted for clinical variables that reached p value of <0.3 in the comparison of patients with and without significant ISR.

Abbreviations: *TGFB1 –transforming growth factor beta 1*, *PDGFB—platelet-derived growth factor beta polypeptide*, *EGF–epidermal growth factor*, *bFGF–basic fibroblast growth factor*, *VEGF-A–vascular endothelial growth factor A*.

### Late lumen loss–multivariable analysis

In the multivariable analysis, LLL was significantly lower in patients with the A/A genotype of rs1800470 (*TGFB1*) versus those with the A/G and G/G genotypes and higher in patients with the A/A genotype of the *VEGF-A* polymorphism versus the A/C and C/C genotypes. The C/C genotype of rs2285094 (*PDGFB*) was associated with higher lumen loss compared to C/T heterozygotes and T/T homozygotes (Table 6).

**Table 6:** Genotype and late lumen loss in the analyzed population.

Gene/Polymorphism	Dominant Model (mean ± standard error)		P	Recessive Model (mean ± standard error)		P	Log additive	P
	Genotypes			Genotypes			Difference per minor allele (95%CI)	
***TGFB1***(rs1800470)	A/A (ref.)	A/G + G/G		A/A + A/G (ref.)	G/G			
	0.495 ± 0.069	0.658 ± 0.054	0.008	0.621 ± 0.049	0.547 ± 0.084	0.59	0.07 (-0.032 ÷ 0.184)	0.17
***PDGFB*** (rs2285094)	T/T (ref.)	T/C + C/C		T/T + T/C (ref.)	C/C			
	0.578 ± 0.067	0.615 ± 0.054	0.14	0.57 ± 0.042	0.80 ± 0.154	0.03	0.127 (0.012 ÷ 0.242)	0.03
***EGF*** (rs4444903)	A/A (ref.)	A/G + G/G		A/A + A/G (ref.)	G/G			
	0.56 ± 0.07	0.623 ± 0.05	0.68	0.576 ± 0.047	0.684 ± 0.092	0.99	-0.013 (-0.116 ÷ 0.089)	0.80
***bFGF*** (rs308395)	C/C (ref.)	C/G + G/G		C/C + C/G (ref.)	G/G			
	0.591 ± 0.046	0.65 ± 0.102	0.48	*	*		*	
***VEGF-A*** (rs699947)	A/A (ref.)	A/C + C/C		A/A + A/C (ref.)	C/C			
	0.678 ± 0.083	0.57 ± 0.049	0.03	0.579 ± 0.047	0.692 ± 0.098	0.47	-0.057 (-0.165 ÷ 0.051)	0.30

* not calculated because of low number of minor homozygotes.

The models were adjusted for angiographic variables that reached p value of <0.3 in the comparison of patients with and without significant ISR.

Abbreviations: *TGFB1 –transforming growth factor beta 1*, *PDGFB—platelet-derived growth factor beta polypeptide*, *EGF–epidermal growth factor*, *bFGF–basic fibroblast growth factor*, *VEGF-A–vascular endothelial growth factor A*.

## Discussion

The migration of SMCs, which constitute more than 90% of neointima cells, from the tunica media through the damaged basal membrane and into the vessel lumen is stimulated by growth factors, mainly TGF-β1, PDGFB, bFGF, EGF and, to a lesser extent, VEGF-A. However, apart from one recent paper focusing on polymorphisms of the *TGFB1* gene (14), genetic polymorphisms of the above growth factors have not been analysed. In view of the fact that at the time of selecting polymorphisms to be analysed there was no studies published regarding the relation of polymorphisms in the genes encoding growth factors to the process of restenosis, polymorphisms analysed in this study were selected on the basis of their functionality. Functionality was understood as the impact on the level of expression of a given gene and/or relation to the concentration of the given growth factor (rs1800470, rs4444903, rs699947) and finally the relation of the specific polymorphism to the occurrence of other conditions or prognosis in other diseases (rs1800470, rs4444903, rs699947, rs308395, rs2285094).

### Rs1800470 (*TGFB1*)

The role of TGF-β1 in restenosis appears to be confirmed, but its underlying mechanism has not been fully explained due to the complex role of TGF-β1. TGF-β1 has an antiproliferative effect because it inhibits cell proliferation at the G1 stage [33]; however, at levels exceeding 1–2 fg/cell, TGF-β1 may stimulate proliferation of SMCs, fibroblasts and chondrocytes [11]. TGF-β1 is secreted locally by arterial wall fibroblasts, where it has a paracrine effect on SMCs, endothelial cells and macrophages migrating to a site injured after PCI. Additionally, platelets participating in parietal thrombus formation after an arterial wall injury release significant quantities of TGF-β1 [34]. TGF-β1 is also found in blood serum, and its levels may also depend on genetic factors. Some researchers have associated the C alleles with higher TGF-β1 levels [35–37], whereas other authors postulated higher levels in T/T homozygotes [38].

However, the role of TGF-β1 in restenosis is not a simple function of its serum levels. In studies conducted at our centre, we did not observe a relationship between TGF-β1 serum levels and restenosis history, recurring restenosis or first restenosis [39,40]. In the first paper on the role of rs1800470, Fragoso et al. demonstrated that, in the Mestizo population, the T allele of rs1800470 is associated with a higher risk of restenosis [14]. Our results for the Polish population indicate that the A allele (the T allele on the complementary strand) is associated with a lower risk of restenosis. However, aside from different ethnic backgrounds, our study group differs from that of Fragoso et al. in the type of implanted stent. In Fragoso et al., patients were implanted with either an antimitotic stent or a metal stent, which may affect the results because the restenosis mechanisms for these two types of stents differ [41]. Ethnicity, the type of implanted stents, and the fact that our population comprised patients with SCAD, whereas the population studied by Fragoso et al. also included patients with acute coronary syndromes, could account for the discrepancy in the relationship between the T and C alleles of rs1800470 and restenosis in the Polish and Mestizo populations [14]. These differences, although difficult to explain, are common in terms of the analysis of the already mentioned relationship between *TGFB1* polymorphisms and TGF-β1 serum levels and between rs1800470 and other cardiovascular diseases. For example, in a population of Italian and German patients, the C/C genotype was associated with a higher risk of myocardial infarction [42,43]. However, Yokota et al. and Cruz et al. demonstrated that, in Japanese and Mexican populations, respectively, the T allele is associated with a higher risk of myocardial infarction [35,44]. In an European population, T allele carriers with rheumatoid arthritis more frequently suffered from hypertension [45]. One study implied a higher risk of IgA nephropathy development in T allele carriers [46], whereas another study did not confirm this relationship [47]. It would be difficult to postulate a mechanism for a role of rs1800470 in restenosis intensification due to the large number of signalling pathways and processes controlled by TGF-β1. However, the role of this polymorphism may depend on other genetic, environmental, or patient-dependent (e.g., severity of coronary artery atherosclerosis) factors, which could also explain the different between the results obtained by Fragoso et al. and our results concerning the relationship between restenosis risk and the T allele of rs1800470.

### Rs2285094 of the *PDGFB* gene

*PDGFB* is a growth factor for cells of mesenchymal origin, including SMCs, and supports their migration to the lumen of an injured vessel. PDGFB is released from platelets forming a parietal thrombus and from activated macrophages migrating to an injury in the arterial wall [48,49]. PDGFB receptor kinase inhibitors inhibit neointima formation in an experimental model [48]. Rs2285094 is located in an intron near a splice site. This polymorphism has been associated with a risk of scleroderma development [17]. No relationship was observed between the risk of type 1 diabetes and IgA nephropathy development and the rs2285094 genotype [15,16]. In our analysis, we observed higher LLL in the C/C homozygote group, even after correction for angiographic variables, suggesting that this result is not an artefact.

### Polymorphisms of the *EGF*, *bFGF* and *VEGF-A* genes

Damage to the layer of SMCs results in the release of preformed growth factors, including bFGF and EGF [50]. Under experimental conditions, mitogen activity in the medium in which SMCs were immersed decreased by 34% following administration of anti-EGF antibodies [50]. EGF may influence restenosis primarily through SMC migration to the vessel lumen because an EGF receptor antagonist inhibited SMC migration but not proliferation [51]. A relationship between rs4444903 and a risk of development and malignancy of numerous neoplastic diseases was demonstrated [20,52,53], thus confirming its “functionality”. Our study is the first to examine the relationship between this polymorphism and restenosis, but no relationship between rs4444903 and the intensity of this process was identified.

bFGF is a growth factor influencing both smooth muscle and endothelial cells. Rs308395 is located within the *bFGF* gene promoter region; thus, it may influence the binding of transcription factors and *bFGF* expression [18,19]. A relationship was observed between this polymorphism and the prognosis of patients with non-Hodgkin lymphoma. Patients with the C/C genotype were more frequently diagnosed with an aggressive form of this disease compared to G allele carriers [19]. Experimental studies have demonstrated that bFGF levels increase in the first hours after balloon angioplasty or stent implantation, which may be related to intensified inflammatory response [54], whereas SMC proliferation after arterial wall injury is inhibited by antibodies directed against bFGF [55]. However, in dogs undergoing iliac artery angioplasty, bFGF infusion restored endothelium-dependent and -independent relaxation of the arterial wall and reduced LLL [56]. As bFGF stimulates endothelial cell proliferation, thereby accelerating stent endothelialisation, bFGF was proposed as a possible substance for stent coating [57]. Similar studies are in progress for VEGF-A, which also stimulates endothelial cell proliferation [58]. Furthermore, higher VEGF-A levels after PCI are related to restenosis in stents coated with an antimitotic drug [5,59]. Rs699947 has been widely studied in autoimmune and neoplastic diseases. Lower levels of this cytokine are observed in individuals with Kawasaki disease and A/A and A/C genotypes [60]. Additionally, a higher risk of colon cancer and osteosarcoma was identified in carriers of the A allele [61]. Relationships have also been identified between *VEGF-A* polymorphisms and the development of collateral circulation in individuals with coronary artery disease [23], the risk of coronary artery disease in the general population [62], and myocardial infarction in patients with rheumatoid arthritis [63]. Although VEGF-A stimulates stent re-endothelialisation and SMC migration and rs699947 has been previously associated with coronary artery disease, our study is the first to examine this polymorphism in terms of restenosis and identify an association with LLL.

### Study strengths and limitations

The strengths of this study include the first (with the exception of the *TGFB1* gene) polymorphism analysis of genes encoding growth factor proteins with confirmed roles in restenosis. Polymorphisms of the *TGFB1*, *VEGF-A*, *bFGF* and *EGF* genes are functional polymorphisms that affect the levels of these growth factors and are associated with numerous, mainly neoplastic, diseases. Another strength of this study is the fact that the number of patients enrolled was sufficient to detect, with a power of 80%, the differences in LLL in a vessel below 0.05 mm, assuming a standard deviation of 0.1 mm under the dominant or recessive model for each polymorphism studied. However, the size of the study group was sufficient to detect a 15% or greater difference in the incidence of significant angiographic restenosis for the dominant/recessive model for all examined polymorphisms which was a limitation of the study. Limitations of this study include also different time to next coronary angiography for patients enrolled, which resulted both from the time of the onset of symptoms and from the various waiting period for an elective procedure, ethnically homogenous study population and small number of homozygotes for the rs308395 polymorphism of the *bFGF* gene.

## Conclusions

The polymorphisms rs1800470, rs2285094 and rs6999447 of the *TGFB1*, *PDGFB* and *VEGF-A* genes, respectively, are associated with LLL in patients with SCAD treated by PCI with a metal stent implantation.
